# Validity and reliability of the measurement instrument of the nursing outcome health-related Physical Fitness (2004), proposed and transculturally adapted to the Spanish context

**DOI:** 10.1186/s12912-022-01121-8

**Published:** 2022-12-03

**Authors:** Jessica Rojas-Navarrete, César Leal-Costa, Gonzalo de La Morena Valenzuela, Isabel Morales-Moreno, Ismael Jiménez-Ruiz, Paloma Echevarría-Pérez

**Affiliations:** 1 Doctoral Program in Health Sciences, Universidad Católica San Antonio de Murcia, RN Hospital Universitario Virgen de La Arrixaca, Murcia, Spain; 2 Faculty of Nursing, Universidad de Murcia, Murcia, Spain; 3 Hospital Universitario Virgen de La Arrixaca de Murcia, Murcia, Spain; 4 Faculty of Nursing, Universidad Católica San Antonio de Murcia, Murcia, Spain

**Keywords:** Nursing outcomes classification terminology, Physical fitness, Health-related physical fitness

## Abstract

**Background:**

Presently, physical inactivity is the main public health problem in many countries worldwide. Physical activity promotes the maintenance or improvement of one’s physical condition. Physical fitness has been established as the main biological marker of the state of health of an individual, and therefore, there is a clear need to measure health-related physical fitness through the use of a reliable and valid instrument. This study is a continuation of the transcultural adaptation process and a new proposal of the nursing outcome Physical Fitness (2004), found in the 5^th^ Edition of the Nursing Outcomes Classification. The objective of this study was to examine the validity and reliability of the nursing outcome Health-Related Physical Fitness survey, proposed and transculturally adapted to the Spanish context.

**Methods:**

An instrumental study to validate the nursing outcome Physical Fitness (2004), from the 5^th^ Edition of the Nursing Outcome Classification was carried out. It took place between the months of May, 2016 to May, 2017. On the first stage, the instrument proposed Health-Related Physical Fitness survey was administered to 160 adults who used the Health Services of Murcia, Spain by three independent evaluators. After 4 weeks, it was administered again to 33 participants to calculate the intra-rater reliability. Lastly, the SF-12v2 Health Survey was administered to obtain external evidence of validity.

**Results:**

The inter-rater reliability of the nursing outcome proposed obtained high values (between 0.91–0.99) in the evaluations performed by the three evaluators. As for the intra-rater reliability, high values were obtained (0.94–1), except for the item “balance”, which was moderate (0.56). Lastly, a positive and statistically significant correlation (*p *< 0.05) was obtained between the Physical Component Summary, and the dimensions Physical Functioning and General Health from the SF-12v2 Health Survey, and the global score of the Health-Related Physical Fitness proposed instrument.

**Conclusions:**

The validity and reliability results of the nursing outcome Health-Related Physical Fitness survey, proposed and transculturally adapted to the Spanish context, were adequate for its use by nurses with adults who use the Health Services of Murcia. However, this instrument must be analyzed with more diverse samples of health services users.

**Supplementary Information:**

The online version contains supplementary material available at 10.1186/s12912-022-01121-8.

## Background

Presently, physical inactivity is the main public health problem in a large number of countries worldwide, as it is a key factor in the development of non-transmissible diseases (NTD) [1, 2], and the fourth highest risk factor for mortality worldwide [3]. The latest available data indicate that approximately 27.5% of the adult population [4] and 81% of adolescents [5] do not perform the physical activity necessary for obtaining health benefits.

Physical fitness (PF) has been established as the main biological marker of the state of health of an individual [6]. PF is “the ability to perform daily-life activities with vigor and care, without excessive fatigue and with enough energy to enjoy the leisure-time activities and face unexpected emergencies” [7]. The components of PF are sub-divided according to two groups: one associated with health, and another associated with the skills related to athletic ability [8]. Health-related physical fitness (HRF) encompasses specific PF components linked with the good state of health of a person and could be determined by regular PA [6]. Nevertheless, the components of the HRF can vary depending on the definition utilized [9]. The main components related with health are cardiorespiratory fitness (CRF), muscle endurance, muscle strength, body composition, and flexibility [10].

HRF is directly and strongly associated with the level of PA of an individual and the maintenance of good health [11, 12]. The use of an instrument to measure HRF can serve as a motivational element for helping individuals increase their levels of PA [13]. The latest WHO guidelines on physical activity and sedentary habits [1] highlights the need for a greater investment on research studies that allow us to evaluate the relationship between PA and health outcomes, and for this to be possible, a precise, simple, and cost-effective instrument is needed for measuring HRF [14]. Many batteries of field tests exist which evaluate the different components of HRF in adult populations, among which we find the Health-Related Fitness Test from the American Alliance for Health, Physical Education, Recreation and Dance (AAHPERD) [15], the Eurofit battery of tests for adults [16], the Canadian Physical Activity, Fitness and LifeStyle Appraisal (CPAFLA) test [17], and the ALPHA-FIT Test Battery for Adults Aged 18–69 [18]. However, there is also a great number of tests that do not allow for the fast assessment of HRF [9], so that their implementation in the area of health is limited to certain requirements, such as time, cost, and the practical experience necessary for their administration [19], as well as the equipment and space resources needed.

The increase in research on standardized nursing languages has significantly contributed to the professional development of nursing [20]. Likewise, in the last few years, an increase in the interest in nursing outcomes has been observed, as they have been shown to play an active role on the quality of the care and profitability of health systems [21]. The most important effort for trying to identify and refine results that are sensitive to the activity of the nurses has been the development of the Nursing Outcomes Classification (NOC) [22, 23]. Presently, one of the great changes in research studies that address outcomes associated to the practice of nursing is linked to the development and validity of instruments that are appropriate for its evaluation [21, 24]. In this sense, the use of validated NOC nursing outcomes allows nurses to efficiently evaluate the health outcomes of users, as well as to determine the effect of the nursing interventions [25]. The nursing outcome Physical Fitness (2004), from the 5^th^ Edition of the NOC [26] consists of a series of indicators that establish a variety of states, behaviors, or perceptions related to PF, and that serve as a guide to evaluate the object of study.

The main contribution of our study is the simplification of the HRF evaluation process through the selection of a series of short-duration field tests (approximately 8–10 min total) that are easy to administer, as neither a large amount of experience, high motivation for their performance, nor sophisticated or costly laboratory equipment, or a large space for their performance, are needed. In this manner, the validity and reliability of the measurement of all the HRF components is guaranteed, in any area of health, either in a nurse consultation, or a hospital floor [27].

The main objective of this study was to analyze the reliability and external validity of the nursing outcome Health-Related Physical Fitness (2004), proposed and transculturally adapted to the Spanish context and the target language, through the selection of a battery of reliable and valid tests for the evaluation of the HRF by nurses in a healthcare context. This study is a continuation of the initial study on the transcultural adaptation process to the Spanish context, acquisition of the internal validity, and a new proposal of the nursing outcome Physical Condition (2004*)* from the 5th Edition of the NOC [26], normally considered to be the first step for the validation of a measurement instrument [28].

## Methods

### Design and participants

An Instrumental study was carried out to obtain evidence of reliability and validity [29] of the nursing outcome Health-Related Physical Fitness (2004), proposed and transculturally adapted to the Spanish context [30].

To obtain evidence of reliability and validity of the proposed nursing outcome, a total of 160 adults who were users of Primary Care services in the Murcia Health Services (Spain) participated in the study. For the selection of the study participants, a convenience sampling method was utilized. The inclusion criteria were being a user of Primary Care services in the Murcia Health Services, and aged between 20 and 69 years old. The exclusion criteria were having some type of medical contraindication for performing PA and/or physical exercise and having high blood pressure (BP) values (systolic blood pressure (SBP) > 150 mmHg and diastolic blood pressure (DBP) > 95 mmHg) at the moment the instrument was administered.

### Procedure

The study was conducted between May, 2016, and May, 2017. The instrument was administered to each study participant by three independent evaluators who had a nursing degree, and who had received basic professional training for its correct use, through a training session which lasted 3 h in two practice sessions. The evaluators knew about the development and objectives of the different tests, as well as the interpretation of the results. Evaluator 1 was used as the model evaluator, and the measurements were always taken following the same order (evaluator 1, evaluator 2, and evaluator 3).

Before administering the test, a series of recommendations were provided to the study participants, based on the Health-Related Physical Fitness Assessment Manual [31] for the evaluation of the HRF, which are detalied here: wear comfortable sports clothing, be well-hydrated, not smoking, no caffeine or any other type of stimulating and/or diuretic substances (i.e. tea, chocolate) 24 h before the evaluation, not having consumed alcohol 48 h prior. Likewise, a recommendation was provided to not perform vigorous physical exercise in the 24 h prior, nor low and /or medium intensity physical exercise 12 h before the evaluation, and sleeping 7–8 h the night before. Also, to measure the body composition, a recommendation was given to empty their bladder 30 min before the test. Then, a signed informed consent form was asked from each user, and a survey was provided to collect their demographic and health data, such as name and last names, date of birth, date of the study, medical history of interest, and pharmacological treatment (in the case that the participant had a prescribed pharmacological regime). Also, their blood pressure was taken at rest to verify the non-contraindication for performing the different tests, after which the SF-12v2 Health Survey [32] was administered to discover how they considered their health, and to analyze Pearson’s Correlation Coefficient (PCC). Lastly, each participant was informed about the specific instructions for the correct execution of each test.

The sequence of field tests administered for the evaluation of each item from the instrument was as follows: the measurement of the waist circumference and percentage body fat with bioelectrical impedance scale, calculation of body mass index, One Leg Stand Test to measure balance, Sit-and-Reach Test to measure flexibility, manual dynamometry to measure muscle strength and a modified Queen´s College step Test to measure cardiorespiratory fitness.

After four weeks, the proposed instrument was administered again to a sample of 33 subjects by the same evaluators, to evaluate the temporal stability of the results.

### Instruments

The instrument utilized was the proposed nursing outcome Health-Related Physical Fitness, which was transculturally adapted to the Spanish context [30]. To measure its different items, various reliable and validated field tests were selected, as detailed below:

#### Cardiorespiratory Fitness

For the evaluation of the CRF, the Queen’s College Step Test (QCT), also known as the McArdle step test [33] was utilized, with one modification. The QCT is a sub-maximal field test utilized to measure CRF based on recovery heart rate (HR). The modified QCT performed was identical to the QCT, with the only variation being that the height of the step was set as the height reached by the foot of the dominant leg of the subject when the knee was bent at a 90° angle. The result of maximal oxygen uptake (VO_2max_) was obtained after introducing the data into the following equations [33]:

$$\mathrm{For}\;\mathrm{men}:\;{\mathrm{VO}}_{2\max}\;(\mathrm{mL}\cdot\mathrm{kg}^{-1}\cdot\min\nolimits^{-1})\:=\:111.33-(0.42\:\times\:\mathrm{HR})$$

$$\mathrm{For}\;\mathrm{women}:\;{\mathrm{VO}}_{2\max}\;(\mathrm{mL}\cdot\mathrm{kg}^{-1}\cdot\min\nolimits^{-1})\:=\:65.81-(0.1847\:\times\:\mathrm{HR})$$

The reference values utilized to categorize the VO_2max_ results into a Likert scale of the nursing outcome Health-Related Physical Fitness were developed by the Cooper Institute in Dallas, Texas [34] (Table S1).

#### Muscle strength

For the quantitative measurement of the muscle strength, the handgrip strength was measured with a Camry® precision electronic dynamometer model EH101, with a maximum capacity of 198 lb/90 kg and an adjustable grip. To perform the test, the procedure described by the American College of Sports Medicine (ACSM) [31] was followed, and to categorize the scores obtained from the manual dynamometer in relation with the body weight in the Likert scale of the nursing outcome Health-Related Physical Fitness (Table S2), the reference guidelines from the Eurofit battery test for adults was utilized [16].

#### Flexibility

The flexibility test utilized was the classic Sit-and-Reach Test (SRT) [35]. The SRT is a trunk flexion test performed the sitting position, which provides a measurement of flexibility of the hamstring muscles, hip, and lower back. For this, a graduated box measuring 32 cm in height and 50 in length was utilized, with a 45 cm-wide horizontal board, and with point 0 located at the 26 cm mark. To categorize the scores obtained in the SRT in the Likert scale of the nursing outcome Health-Related Physical Fitness, the guideline values from the Canadian test were utilized [36] (Table S3).

#### Balance

The test utilized to measure static balance was the One Leg Stand Test (OLST) [18]. Each participant had to maintain balance on one leg for a maximum time of 60 s. To categorize the scores obtained in the OLST in the Likert scale of the nursing outcome Health-Related Physical Fitness, the guidelines from the ALPHA-FIT Test Battery for Adults Aged 18–69 [18] was utilized (Table S4).

#### Waist circumference

To evaluate the waist circumference (WC), a highly precise, flexible and inelastic ergonomic Seca® band, with automatic winding for measuring circumferences was used. To categorize the scores obtained in the WC in the Likert scale of the nursing outcome Health-Related Physical Fitness, the classification established by the WHO for the WC specific to sex and the risk of metabolic complications associated with obesity in Caucasians was utilized [37] (Table S5).

#### Body weight and percentage of body fat

The measurement of body weight and the percentage body fat was performed by utilizing a bioelectrical impedance scale, Tanita® model BF-350 (precision of 100 g; range 0–150 kg). The reference values utilized to categorize the percentage body fat into a Likert scale of the nursing outcome Health-Related Physical Fitness were developed by the Cooper Institute in Dallas, Texas [34] (Table S6).

#### Height

Height was measured with a portable height stadiometer, a Tanita® model HR001, composed of a graduated vertical column (with a precision of 0.1 cm; range from 0–207 cm), and a horizontal platform.

#### Body mass index

The calculation of the body mass index (BMI), also called the Quetelet index, was performed through the internationally-accepted formula: weight (kg)/height^2^ (m). To categorize the scores obtained for the BMI in the Likert scale of the nursing outcome Health-Related Physical Fitness, the classification established by the WHO was utilized [37] (Table S7).

#### Blood pressure

An Omron® automated digital monitor model M10-IT was utilized to measure the resting BP. The new American College of Cardiology high blood pressure guidelines establish five categories to define hypertension. In this category, stage 2 is established when we find a systolic blood pressure (SBP) of at least 140, or a diastolic blood pressure (DBP) of at least 90 mmHg. According to this classification, in our study, we considered including people with SBP values lower than 150, and DBP values lower than 95 mmHg [38].

#### SF-12v2 Health Survey

To evaluate the health-related quality of life (HRQoL), the Spanish version of the SF-12v2 Health Survey was utilized [39, 40].

The SF-12 Health Survey is a widely-used instrument for the evaluation of HRQoL [32, 41]. It is the abridged version of the SF-36 Health Survey [32]. The SF-12v2 includes 12 items from the 8 total dimensions of the original SF-36: 1) Physical Functioning (PF, 2 items); 2) Role-Physical (RP, 2 items); 3) Bodily Pain (BP, 1 item); 4) General Health (GH, 1 item); 5) Vitality (VT, 1 item); 6) Social Functioning (SF, 1 item); 7) Role-Emotional (RE, 2 items); and 8) Mental health (MH, 2 items). These 8 items are encompassed into 2 components: The Physical Component Summary (PCS), which includes dimensions PF, RP, BP, and GH, and the Mental Component Summary (MCS), which includes the dimensions VT, SF, RE, and MH [42]. The SF-12v2 scores range from 0 to 100, with higher scores indicating a better HRQoL. The PCS (range 0–100) and the MCS (range 0–100) scores were also calculated through the sum of the dimensions that comprised each summary component of the HRQoL [43]. To calculate the scores, the standard American calculation algorithm was utilized through the QualityMetric Health Outcomes™ Scoring software 5.0.

### Data analysis

The statistical analysis of the data was performed using the statistical program IBM SPSS Statistics 27. The inter-rater and intra-rater reliability was calculated through the intraclass correlation coefficient (ICC). The inter-rater reliability was calculated in 6 of the 7 items of the instrument, as the item percentage of body fat was only measured once with a bioelectrical impedance scale. However, the intra-rater reliability [44] was calculated in all 7 items of the instrument. The ICC was calculated using a random model of 2 factors and absolute agreement to analyze the degree of agreement between the scores of the different items of the instrument obtained for each of the three observers (inter-rater reliability), and to analyze the test–retest results (intra-rater reliability). Unique measurements were obtained with a confidence interval (CI) of 95%. The level of statistical significance was *p* < 0.05. The interpretation of the ICC was performed according to following classification [45], where 0.75–1.00 indicated a very good result; 0.60–0.74 a good result; 0.40–0.59 a moderate result; with < 0.40 indicating a bad result.

Lastly, to obtain external evidence of validity, the Pearson’s bivariate correlations were calculated between the two dimensions (PCS and MCS) of the SF-12v2 Health Survey, and the total score from the nursing outcomes Health-Related Physical Fitness.

### Ethical considerations

This study was conducted by following the guidelines for good clinical practice [46]. The authorization for the study was obtained from the Ethics Committee of Clinical Research from the University Hospital Virgen de la Arrixaca (Murcia, Spain), with internal code: 2015–12-8-HCUVA. The informed consent was acquired from each of the users for their voluntary participation in the study.

## Results

A total of 160 users of the first level of care from Health Area 1 of the public health service from the Region of Murcia (61, 38.1% men; 99, 61.9% women), with an age range between 20 and 69 years old (M = 45.15 (SD = 13.50)), participated in the study.

Table 1 shows the descriptive characteristics (mean (SD)) of the study sample according to sex. In general terms, the men had higher a CRF (54.0 as compared to 39.1), muscle strength (49.2 as compared to 26.8), and balance (58 as compared to 54). On the other hand, the women had a greater flexibility (25.5 as compared to 22.8). As for the body composition, the men had higher values of BMI (27.3 as compared to 26.4), and WC (93.0 as compared to 81.4). However, the percentage of body fat was greater in women (33.2 as compared to 21.7). These gender differences in fitness level were statistically significant (*p* < 0.05) for all the components of HRPF except for the age, BMI, and flexibility. In addition, the gender differences in relation to the components from the SF-12v2 were also statistically significant (*p* < 0.05) for all physical components except for the dimensions VT and RE included in the MCS.

**Table 1:** Descriptive characteristics of the study sample according to sex

	Total (*n* = 160)	Men (*n* = 61)	Women (*n* = 99)	*p*
Age (years)	46.15(13,50)	46.62(13.57)	45.87(13.52)	0.734
SBP (mmHg)	123(16)	128(15)	120(15)	0.001
DBP (mmHg)	78(9)	80(10)	76(9)	0.004
Weight (kg)	73.2(14.2)	82.8(13.1)	67.3(11.5)	< 0.001
Height (cm)	165.2(9.5)	173.9(7.0)	159.9(6.4)	< 0.001
BMI (kg/m^2^)	26.78(4.53)	27.37(3.98)	26.42(4.81)	0.200
Percentage body fat	28.8(9.9)	21.7(6.9)	33.2(8.9)	< 0.001
WC (cm)	85.8(12.4)	93.0(10.7)	81.4(11.3)	< 0.001
Balance (s)	55(12)	58(7)	54(13)	0.025
Flexibility (cm)	24.5(9.5)	22.8(9.1)	25.5(9.7)	0.082
Muscle strength (kg)	35.3(12.9)	49.2(9.5)	26.8(4.4)	< 0.001
Muscle strength (N/kg)	4.8(1.5)	6.1(1.4)	4.1(0.9)	< 0.001
CRF (VO_2max_, mL·kg^−1^·min^−1^)	44.90(9.46)	54.05(8.05)	39.10(4.28)	< 0.001
HRQoL (SF-12v2)^a^				
Physical Functioning (PF)	52.44(7.35)	54.74(5.25)	51.02(8.09)	0.002
Role-Physical (RP)	50.03(9.33)	52.19(7.63)	48.70(10.05)	0.021
Bodily Pain (BP)	51.42(8.33)	53.44(7.83)	50.17(8.42)	0.015
General Health (GH)	48.63(9.47)	50.65(8.77)	47.39(9.72)	0.034
Vitality (VT)	53.34(9.23)	55.13(9.60)	52.25(8.87)	0.056
Social Functioning (SF)	50.08(9.24)	52.45(7.04)	48.64(10.11)	0.011
Role-Emotional (RE)	45.83(11.98)	48.99(10.95)	43.89(12.22)	0.08
Mental Health (MH)	49.38(10.11)	52.83(9.48)	47.29(9.95)	0.01
Physical Component Summary (PCS)	52.23(7.22)	53.89(6.48)	51.23(7.48)	0.024
Mental Component Summary (MCS)	47.98(10.48)	51.00(10.04)	46.14(10.36)	0.04

The results shown are mean and SD

*Abbreviations*: *SBP* systolic blood pressure, *DBP* diastolic blood pressure, *BMI* body mass index, *WC* waist circumference, *CRF* cardiorespiratory fitness, *HRQoL* Health related quality of life

^a^High scores indicate a better HRQoL

Table 2 shows the descriptive analysis of the results obtained after administering the proposed instrument Health-Related Physical Fitness categorized with the Likert scale of the NOC, in which a value of 1 indicated the worst result possible, and 5 the best one. The results showed that the level of HRF in general was good, with a good mean score of (M = 3.36) in the Likert scale. As for the different items in the measurement instrument, the components that received the highest scores were CRF (M = 4.45) and balance (M = 4.60), which indicates that the population studied had a good aerobic capacity and balance. The mean score of the BMI was M = 4.06, which shows that the mean population was overweight. The WC obtained a mean score of M = 3.47, indicating a moderate risk of metabolic complications associated with obesity. Lastly, the items that obtained the lowest scores were muscle strength (M = 2.53), flexibility (M = 2.24), and percentage body fat (M = 2.34), which suggests that the musculoskeletal system and percentage body fat were the most deficient components of the HRF in the study subjects.

**Table 2:** Descriptive characteristics of the categorized scores of the nursing outcome Health-Related Physical Fitness

	Global score	CRF	MS	Flexibility	Balance	BMI	WC	% Fat mass
N								
Valid	160	152	160	160	160	160	160	160
Lost	0	8	0	0	0	0	0	0
Mean	3.36	4.45	2.53	2.24	4.60	4.06	3.47	2.34
Median	3.42	4.60	2.31	1.90	4.76	4.17	4.07	1.92
Mode	3	5	2	1	5	4	5	1
Percentiles								
25	2.82	4.02	1.41	1.16	4.19	3.36	1.78	1.12
50	3.42	4.60	2.31	1.90	4.76	4.17	4.07	1.92
75	4.00		3.53	3.17		4.83	4.93	3.58

The scores are based on the Likert scale of the proposed nursing outcomes Health-Related Physical Fitness

*Abbreviations*: *CRF* cardiorespiratory fitness, *MS* muscle strength, *BMI* body mass index, *WC* waist circumference, %, Percentage

Table 3 shows the means and SD of the dimensions and summary components of the SF-12v2 Health Survey with scores from 0 to 100. The means of the dimensions were found to be between 45.8 for GH and 53.5 for VT. The SD were relatively low, with values ranging from 7.22 for PCS, to 11.98 for the RE dimension. The highest scores (> 60) were obtained in the dimensions GH, VT, MH, and in the mental and physical health measures of HRQoL. In general terms, the men (Table 1) had a greater HRQoL as compared to the women, with higher scores found in the PCS (53.8 as compared to 51.2), as well as in the MCS (51.0 as compared to 46.1).

**Table 3:** Means and standard deviations of dimensions and summary components of the SF-12v2 Health Survey

	N	Mean^a^	SD	Range	Minimum	Maximum
Physical Functioning (PF)	160	52.44	7.352	0–100	25.58	57.06
Role-Physical (RP)	160	50.03	9.33	0–100	23.61	57,46
Bodily Pain (BP)	160	51.42	8.33	0–100	21.66	57.73
General Health (GH)	160	48.63	9.47	0–100	23.90	63.66
Vitality (VT)	159	53.34	9.23	0–100	29.39	68.74
Social Functioning (SF)	159	50.08	9.24	0–100	21.32	56.90
Role Emotional (RE)	160	45.83	11.98	0–100	14.70	58.49
Mental Health (MH)	159	49.38	10.11	0–100	18.32	64.21
Physical Component Summary (PCS)	159	52.23	7.22	0–100	31.37	66.90
Mental Component Summary (MCS)	159	47.98	10.48	0–100	20.54	65.75

^a^Calculation of the mean and standard deviation (SD) of the sample through the original (or standard) American calculation algorithm

### Reliability analysis

The results of the study indicated adequate inter-rater and intra-rater reliability for the overall result and for each of the items of the proposed instrument that was transculturally adapted to the Spanish context. Table 4 shows the results of the inter-rater reliability: 0.99 for the general score of the instrument, and for the rest of the items, the following results were obtained: cardiorespiratory fitness: 0.98; muscle strength: 0.99; flexibility: 0.99; balance: 0.94; body mass index: 0.91; and waist circumference: 0.95.

**Table 4:** Inter-rater reliability of the proposed and transculturally adapted nursing outcome Health-Related Physical Fitness

Nursing outcome Health-Related Physical Fitness and items	ICC	95% CI	*p*
Global score	0.99	0.99–0.99	< 0.001
Cardiorespiratory fitness	0.98	0.97–0.98	< 0.001
Muscle strength	0.99	0.99–0.99	< 0.001
Flexibility	0.99	0.99–0.99	< 0.001
Balance	0.94	0.92–0.95	< 0.001
Body mass index	0.91	0.88–0.93	< 0.001
Waist circumference	0.95	0.94–0.96	< 0.001

Table 5 shows a high degree of agreement between the test and re-test scores, which points to the stability of the nursing outcome and the items through time. The result obtained for the global score of the instrument was 0.96, and for each of the items of the proposed instrument, the following scores were obtained: cardiorespiratory fitness: 0.96; muscle strength: 0.98; flexibility: 0.95; balance: 0.56; body mass index: 1.00; waist circumference: 0.99; and percentage body fat: 0.94.

**Table 5:** Intra-rater reliability of the proposed and transculturally adapted nursing outcome Health-Related Physical Fitness

Nursing outcome Health-Related Physical Fitness and items	ICC	95% IC	*p*
Global score	0.96	0.92–0.98	< 0.001
Cardiorespiratory fitness	0.96	0.91–0.98	< 0.001
Muscle strength	0.98	0.96–0.99	< 0.001
Flexibility	0.95	0.90–0.97	< 0.001
Balance	0.56	0.11–0.78	0.012
Body mass index	1.00	1.00–1.00	< 0.001
Waist circumference	0.99	0.97–1.00	< 0.001
Percentage body fat	0.94	0.84–0.97	< 0.000

### External validity analysis

The results of the analysis of the bivariate correlations between the summary measures (PCS and MCS) and the dimensions (PF, RP, GH, VT, SF, MH, and RE) of the Spanish version of the SF-12v2 Health Survey, and the total score of the nursing outcome proposed Health-Related Physical Fitness, and its items (CRF, muscle strength, flexibility, balance, BMI, WC, and percentage body fat), are shown in Table 6. The PCS had a significant positive association (*p* < 0.01) with the global score of the nursing outcome and the items muscle strength, flexibility, and balance. However, the PCS was negatively associated (*p* < 0.01) with the items that constituted body composition (BMI, WC, and percentage body fat). On the contrary, the PCS did not have a significant association with the CRF component. The MCS did not show significant associations (*p* > 0.05) with the overall score of the nursing outcome or its items. On the other hand, the PF, RP, and GH scales of the SF-12v2 Health Survey had the greatest positive association (*p* < 0.01) with the overall score of the nursing outcome.

**Table 6:** Bivariate correlations between dimensions and components of the SF-12v2 with the outcome Health-Related Physical Fitness

	PCS	MCS	PF	RP	BP	GH	VT	SF	RE	MH
CRF	0.111	0.158	0.126	0.141	0.126^*^	0.077	0.039	0.183^*^	0.137	0.191
Muscle strength	0.294^**^	0.177^*^	0.319^**^	0.228^**^	0.175^*^	0.321^**^	0.164^*^	0.214^**^	0.180^*^	0.209^**^
Flexibility	0.290^**^	0.104	0.227^**^	0.206^**^	0.186^*^	0.262^**^	0.157^*^	0.215^**^	0.123	0.084
Balance	0.359^**^	0.109	0.363^**^	0.230^**^	0.257^**^	0.270^**^	0.198^*^	0.129	0.175^*^	0.127
BMI	-0.261^**^	0.014	-0.250^**^	-0.153	-0.002	-0.301^**^	-0.066	-0.068	-0.035	0.008
WC	-0.216^**^	0.076	-0.167^*^	-0.095	0.052	-0.278^**^	-0.040	-0.012	0.051	0.069
Percentage fat mass	-0.385^**^	-0.146	-0.414^**^	-0.272^**^	-0.178^*^	-0.399^**^	-0.155	-0.222^**^	-0.149	-0.214^**^
HRF Global score	0.436^**^	0.116	0.377^**^	0.323^**^	0.230^**^	0.404^**^	0.197^*^	0.207^**^	0.161^*^	0.137

The data are shown as the Pearson's correlation coefficient. ^*^ *p* < 0.05,^**^p < 0.01

*Abbreviations*: *PCS* physical component summary, *MCS* mental component summary, *PF* physical functioning, *RP* role-physical, *BP* bodily pain, *GH* general health, *VT* vitality, *SF* social functioning, *RE* role-emotional, *MH* mental health, *CRF* cardiorespiratory fitness, *BMI* body mass index, *WC* waist circumference, *HRF* health-related physical fitness

## Discussion

The results obtained in our study showed a greater CRF in men than in women. These results are in agreement with those obtained in the study by Varghese et al. [47], in which the administration of the QCT to 501 adult Indians indicated a greater mean VO_2max_ values in men, and the study by Hoffmann et al. [48], which also concluded that the CRF measured through a modified Canadian Aerobic Fitness Test (mCAFT) sub-maximal step test was also greater in men, thus establishing a statistically-significant association between gender and VO_2max_. Likewise, the mean VO_2max_ values found in the present work were also higher, in agreement with the study mentioned [47] and the study by John et al. [49], which showed that the VO_2max_ of the Caucasian population was significantly higher than in the Indian population.

The muscle strength results (measured with the Camry manual dynamometer) obtained by our sample also revealed higher mean values in men than in women. These findings are similar to those reported by Sánchez-Torralvo et al. [50], which determined the normative values of dominant hand grip strength in a general sample of the Spanish population through the use of Jamar and Collin dynamometers. Likewise, our findings were consistent with those found by Mateo-Lázaro et al. [51], who obtained higher mean values of grip strength in an adult male population in Teruel (Spain). Other studies have also shown a significant association between grip strength and gender in adults [48, 52, 53].

The results from this study notably showed higher scores for women in the SRT flexibility test with respect to men. At present, lower back and the hamstrings flexibility reference values do not exist for the adult Spanish population. However, our results are backed by previous studies, which showed that women were generally more flexible than men [48, 54]. As for static balance, the result from our study sample showed a good balance in the OLST, which was slightly higher in men with respect to women.

As for the results on the body composition of our study sample, high mean values were obtained for BMI, which were indicative of overweightness, with a greater prevalence in men. Our results coincide with those obtained by López-Sobaler et al. [55], in which more than half of the studied population (Spanish adults aged between 18 and 64 years old) had an excessive weight (BMI > 25 kg/m^2^), with a greater percentage being men. Also, we found a low mean risk of metabolic complications associated with obesity through the evaluation of the WC. These findings are in agreement with the tendency of the Spanish adult population to increase the prevalence of abdonimal obesity, along with overweightness and general obesity [55].

In our study, the test–retest reliability of the QCT indicated a high reproducibility, with scores that were even higher than those obtained by McArdle et al. [33]. Likewise, recent studies on the validity of the QCT have been conducted with Indian youth and adults [47, 56, 57], and corroborate that the QCT is a valid method for indirectly evaluating CRF, as it has a high and statistically significant correlation (*p* < 0.001) between the HR recorded in the QCT and the VO_2max_ measured directly. However, the height of the step can have an influence on the biomechanical efficiency and the HR, so that it has been established that adapting the step to the height of the subject in the step test could more precisely predict CRF [58–62], as muscle fatigue could appear in the legs before we can correctly measure a reliable CRF [59, 63, 64]. The individual adaptation of the height of the step to each of the 160 subjects who participated in the step test showed a high correlation between VO_2max_ and the HR measured with a fingertip pulse oximeter 5-20 s after the QCT, with these results being very similar to those obtained by McArdle et al. [33]. Asley et al. [58] did not find statistically significant differences (*p* < 0.05) between the HR measured with the traditional method of palpation of the radial artery or through a HR monitor, so the method used in our study to measure the HR provides us with a valid and practical method for monitoring the HR in the use of the modified QCT.

As for the measurement of the muscle strength, and as backed by the College of Sports Medicine [65], there is no single universal measurement that provides a complete evaluation of an individual. In spite of this, the grip strength is a well-established biomarker of the state of health and the overall muscle strength in healthy individuals and in adults with pathologies [66], particularly older adults, as it possesses a good clinical value and forecasting power associated to health results [67]. Likewise, grip strength is the simplest method of evaluating muscle strength in clinical practice [68]. Presently, the Jamar dynamometer is the most common device utilized and recommended for measuring grip strength as compared to the Collin dynamometer, because its use could be more precise, as it facilitates grip and use of force [50]. Nevertheless, close values have been obtained (*r* > 0.8; *p* < 0.001) between the use of the Jamar and Camry dynamometers, with the use of the latter being adequate for medical use [69]. Despite the lack of previous studies on the intra-rater reliability with the Camry dynamometer, the high test–retest correlation obtained in the present study coincides with the study by Hogrel [70], in which an excellent reliability was demonstrated (ICC = 0.967) using an electronic manual dynamometer (Myogrip; Ateliers Laumonier, France), which is very similar to the Camry dynamometer.

Flexibility is another important component of HRF, as an inadequate flexibility limits the performance of basic activities of daily living [31]. The classical SRT conducted in our study is based on longitudinal measurements, and is one of the most-commonly used battery of tests in HRF [21, 22, 36, 71] to evaluate the flexibility of the hamstring muscles and the lower back, as it is fast and easy, it requires little practical experience, and can be performed in the field [72]. Also, according to the meta-analysis conducted by Mayorga-Vega et al. [73], the classic version of the SRT has the highest validity for measuring the extensibility of the hamstring muscles. The high intra-rater reliability (0.90–0.97) of the classic SRT obtained in our study agrees with other studies [72, 74–77], independently of the protocol utilized and the sex of the sample. As for the inter-rater reliability, despite the low number of studies analyzed, Gabbe et al. [78] reported an ICC of 0.97 for the classic SRT, with these results also in agreement with those obtained in the present research study.

As for the reliability of the OLST with eyes open, results similar to the study by Suni et al. [26] were obtained, in which the inter-rater reliability of the test was very high (0.76–1.0), as opposed to the same test with the eyes closed or head turned, whose results showed a very poor inter-rater reliability (0.18 and 0.28, respectively). The intra-rater reliability results in our study were moderate, coinciding with those obtained in the study cited [26], in which a moderate intra-rater variability was observed, although it utilized a different statistical measurement (coefficient of variation; CV) to analyze the test–retest reliability. Stones and Kozma [79] also showed moderate values for the intra-rater reliability of the open eyes OLST (ICC = 0.68), concluding that it is a valid and sensitive test for its use in clinical practice and research.

The results from our study showed, as expected, a greater correlation of the overall score of the instrument Health-Related Physical Fitness, with the PCS of the SF-12v2 Health Survey and the dimensions PF, RP, and GH found in this summary component. These results are in agreement with those published by other authors, which provided evidence of a positive association between the PCS and the level of physical fitness measured objectively in university students [80–82] and adults [83–85]. Our results also coincide with other studies, which reported that a greater perceived physical fitness [86] and a high HRQoL [87] were associated with high levels of specific components of physical fitness.

In our sample, the HRF components balance, muscle strength, and flexibility, showed the greatest association with the PCS from the SF-12v2, with the balance component having a stronger association with most of the SF-12v2 dimensions. On the other hand, the BMI, WC, and percentage of body fat had a negative association with the PCS from the SF-12v2, which is similar to the results obtained in the study by Martín-Espinosa et al. [80]. These results also coincide with other international studies, which have provided evidence of a strong negative association between the body composition and the HRF of young adults [88, 89] and middle-aged, and older adults [90–92], thus explaining the inverse relationships obtained in this study between the components of body composition and HRF. The percentage of body fat had a greater negative association with the HRF, also coinciding with the study by Mattila et al. [88], in which the percentage body fat was the strongest predictor of the CRF and the muscle strength in a group of young adults. The CRF did not show any significant relationships with any of the SF-12v2 dimensions in our study.

In the present study, the muscle strength (measured through the use of a dynamometer), was positively associated with both summary components of the SF-12v2 (PCS and MCS) and with all their dimensions. Many studies have reported that grip strength can be considered a good indicator of the PCS and MCS of the HRQoL [93, 94]. However, and despite the limited number of studies that associate the mental dimension of the SF-12v2 and the physical fitness of adults, our results are in agreement with the study by Martín-Espinosa et al. [80], which associated high levels of grip strength in a group of Spanish university students, with a high MCS after administering the SF-12 Health Survey. Likewise, Laredo-Aguilera et al. [95], although with a different HRQoL questionnaire, also revealed a positive association in a sample of women older than 65 in Andalusia (Spain), between grip strength and psychological functioning. However, Gavilán-Carrera et al. [84] reported a lack of association between physical fitness and the MCS from the SF-36 Health Survey or any of its dimensions, in a sample of Spanish women suffering from systemic Lupus eritematoso. In line with these results, although with samples that are not comparable, we find the study by Åvitsland et al. [96], where no significant associations were observed between mental illnesses and some components of the HRF, such as muscle strength and BMI in adolescents.

The lack of association obtained in our sample between the global score of the nursing outcome proposed and the MCS, could be due to the lack of proof of the positive effect of good physical fitness on the promotion of important aspects of mental health, as this involves a series of complex biological mechanisms. Thus, more research studies are needed to show the factors related with the influence of physical fitness on the MCS of the HRQoL [80].

As for the scores of the dimensions and the summary components obtained in the SF-12v2, the men showed a better HRQoL as compared to the women, as observed in other studies [97, 98]. Our results coincide with the reference guidelines of the SF-12v2 Health Survey based on the general population of Catalonia (Spain), in the scores found in the dimensions PF, RP, VT, and SF, as they had the highest means (> 50), and the dimension GH, as it obtained the lowest scores (< 50). However, our sample showed higher values in the PCS as compared to the MCS, in disagreement with the existing normative values in the Spanish population, for which greater MCS scores were reported [40].

### Limitations

Among the main limitations observed in our study, it is necessary to mention that although the SF-12v2 questionnaire was administered to discover the HRQoL of the subjects participating in the study, with a positive correlation obtained with the results from the nursing outcome Health-Related Physical Fitness (2004), the evaluation of the level of PA in the study population through the use of questionnaires could have been complementary data that is easily correlated with the HRF and with the HRQoL, which would have provided additional information of great clinical utility [99, 100].

## Conclusions

This study shows the validity and reliability of the proposed and transculturally adapted to the Spanish population measurement instrument of the nursing outcome Health-Related Physical Fitness, for its use by nurses in a health care environment.

The validated and easy-to-use measurements and field tests provided for measuring each item of the proposed nursing outcome Health-Related Physical Fitness allow nurses to comprehensively assess the HRF of an individual, family, or community, which could also facilitate the proper planning and implementation of nursing care, as well as adequate monitoring of the health status it represents.

The field tests utilized for the measurement of the different items of the proposed instrument are viable in the clinical practice of nurses, and show reliable results for the components of the nursing outcome Health-Related Physical Fitness.

Our results show the adequate reliability and validity of the proposed and cross-culturally adapted to the Spanish context nursing outcome, so the suggested measurement instrument to evaluate HRPF is reliable, safe, and valid for use by nurses in the adult population and in any healthcare setting.

It is therefore essential to continue conducting research for the improvement of the practice of nursing, with special interest on the use of taxonomies, whose demand encompasses the refinement of the components of the nursing diagnoses, interventions, and outcomes.

## Supplementary Information

**Additional file 1: Table S1. **Categories of cardiorespiratory fitness considering the maximal oxygen uptake according to age and sex. **Table S2.** Categories of muscle strength according to manual dynamometer values in relation to body weight (Newtons/kg). **Table S3.** Categories of trunk forward flexibility (cm)* using the Sit-and-Reach Test according to age and sex. **Table S4.** Categories of balance with the use of the One Leg Stand Test according to time (s). **Table S5.** Categories of the waist circumference results (cm). **Table S6.** Categories of percentage of body fat according to age and sex. **Table S7.** Categorization of body mass index.

## Data Availability

The datasets used and/or analyzed during the current study available from the corresponding author on reasonable request.

## Abbreviations

AAHPERD: American Alliance for Health, Physical Education, Recreation and Dance
ACSM: American College of Sports Medicine
BMI: Body mass index
BP: Blood pressure
CPAFLA: Canadian Physical Activity, Fitness and LifeStyle Appraisal
CRF: Cardiorespiratory fitness
GH: General Health
HR: Heart rate
HRF: Health-related physical fitness
HRQoL: Health-related quality of life
ICC: Intraclass correlation coefficient
mCAFT: Modified Canadian Aerobic Fitness Test (sub-maximal step test)
MCS: Mental Component Summary
MH: Mental health
NOC: Nursing Outcomes Classification
NTD: Non-transmissible diseases
OLST: One Leg Stand Test
PA: Physical activity
PCS: Physical Component Summary
PF: Physical fitness
PF: Physical Functioning
QCT: Queen’s College Step Test
RE: Role-Emotional
RP: Role-Physical
SBP: Systolic blood pressure
SF: Social Functioning
SRT: Sit-and-Reach Test
VO_2max_: Maximal oxygen uptake
VT: Vitality
WC: Waist circumference
WHO: World Health Organization
