# Supplementary material for: Validity and reliability of the measurement instrument of the nursing outcome health-related Physical Fitness (2004), proposed and transculturally adapted to the Spanish context
Source: BMC Nurs. 2022 Dec 3;21:340. doi: 10.1186/s12912-022-01121-8 (PMC9719198; doi:10.1186/s12912-022-01121-8)
Supplement: Supplementary file 1 — Additional file 1: Table S1. Categories of cardiorespiratory fitness considering the maximal oxygen uptake according to age and sex. Table S2. Categories of muscle strength according to manual dynamometer values in relation to body weight (Newtons/kg). Table S3. Categories of trunk forward flexibility (cm)* using the Sit-and-Reach Test according to age and sex. Table S4. Categories of balance with the use of the One Leg Stand Test according to time (s). Table S5. Categories of the waist circumference results (cm). Table S6. Categories of percentage of body fat according to age and sex. Table S7. Categorization of body mass index. [file 12912_2022_1121_MOESM1_ESM.docx]

**Supplementary material**

**Table S1. Categories of cardiorespiratory fitness considering the maximal oxygen uptake according to age and sex.**

| Gender | Age range (years) | | | | | Classification | | Likert scale |
| --- | --- | --- | --- | --- | --- | --- | --- | --- |
|  | 20-29 | 30-39 | 40-49 | 50-59 | 60-69 |  |  | |
| M | <36.8 | <35.3 | <33.9 | <31.0 | <27.5 | Very deficient | 1 | |
| W | <31.0 | <29.6 | <28.3 | <26.0 | <24.1 |  |  |  |
| M | 36.8-41.0 | 35.3-39.5 | 33.9-37.7 | 31.0-35.0 | 27.5-31.6 | Deficient | 2 | |
| W | 31.0-35.2 | 29.6-33.8 | 28.3-32.3 | 26.0-29.5 | 24.1-26.8 |  |  |  |
| M | 41.1-44.6 | 39.6-43.9 | 37.8-41.0 | 35.1-38.1 | 31.7-34.9 | Regular | 3 | |
| W | 35.3-38.5 | 33.9-37.1 | 32.4-35.2 | 29.6- 32.3 | 26.9-29.4 |  |  |  |
| M | 44.7-48.4 | 44.0-47.0 | 41.1-44.9 | 38.2–41.9 | 35.0-38.3 | Good | 4 | |
| W | 38.6-42.4 | 37.2-41.0 | 35.3-39.2 | 32.4- 35.3 | 29.5-32.3 |  |  |  |
| M | >48.4 | >47.0 | >44.9 | >41.9 | >38.3 | Excellent | 5 | |
| W | >42.4 | >41.0 | >39.2 | >35.3 | >32.3 |  |  |  |

Abbreviations: M, Man; W, Woman.

**Table S2. Categories of muscle strength according to manual dynamometer values in relation to body weight (Newtons/kg).**

| Gender | Age range (years) | | | | | Classification | Likert scale |
| --- | --- | --- | --- | --- | --- | --- | --- |
|  | 20-29 | 30-39 | 40-49 | 50-59 | 60-69 |  |  |
| M | ≤5.8 | ≤5.5 | ≤5.6 | ≤4.9 | ≤4.9 | Needs improvement | **1** |
| W | ≤4.1 | ≤3.9 | ≤4.0 | ≤3.4 | ≤3.1 |  |  |
| M | 5.9-6.6 | 5.6-6.4 | 5.7-6.1 | 5.0-5.8 | 5.0-5.4 | Average | **2** |
| W | 4.2-4.7 | 4.0-4.7 | 4.1-4.5 | 3.5-3.9 | 3.2-3.6 |  |  |
| M | 6.7-7.2 | 6.5-7.0 | 6.2-6.6 | 5.9-6.4 | 5.5-6.0 | Good | **3** |
| W | 4.8-5.2 | 4.8-5.1 | 4.6-5.0 | 4.0-4.5 | 3.7-3.9 |  |  |
| M | 73-7.9 | 7.1-7.8 | 6.7-7.6 | 6.5-7.0 | 6.1-6.6 | Very good | **4** |
| W | 5.3-5.9 | 5.2-5.7 | 5.1-5.6 | 4.6-5.1 | 4.0-4.6 |  |  |
| M | ≥8.0 | ≥7.9 | ≥7.7 | ≥7.1 | ≥6.7 | Excellent | **5** |
| W | ≥6.0 | ≥5.8 | ≥5.7 | ≥5.2 | ≥4.7 |  |  |

Abbreviations: M, Man; W, Woman.

**Table S3. Categories of trunk forward flexibility (cm)^*^ using the Sit-and-Reach Test according to age and sex.**

| Gender | Age range (years) | | | | | | Classification | Likert scale |
| --- | --- | --- | --- | --- | --- | --- | --- | --- |
|  | 15-19 | 20-29 | 30-39 | 40-49 | 50-59 | 60-69 |  |  |
| H | ≤23 | ≤24 | ≤22 | ≤17 | ≤15 | ≤14 | Needs improvement | 1 |
| M | ≤28 | ≤27 | ≤26 | ≤24 | ≤24 | ≤22 |  |  |
| H | 24-28 | 25-29 | 23-27 | 18-23 | 16-23 | 15-19 | Average | 2 |
| M | 29-33 | 28-32 | 27-31 | 25-29 | 25-29 | 23-26 |  |  |
| H | 29-33 | 30-33 | 28-32 | 24-28 | 24-27 | 20-24 | Good | 3 |
| M | 34-37 | 33-36 | 32-35 | 30-33 | 30-32 | 27-30 |  |  |
| H | 34-38 | 34-39 | 33-37 | 29-34 | 28-34 | 25-32 | Very good | 4 |
| M | 38-42 | 37-40 | 36-40 | 34-37 | 33-38 | 31-34 |  |  |
| H | ≥39 | ≥40 | ≥38 | ≥35 | ≥35 | ≥33 | Excellent | 5 |
| M | ≥43 | ≥41 | ≥41 | ≥38 | ≥39 | ≥35 |  |  |

Abbreviations: M, Man; W, Woman. ^*^These values are based on the SRT box, in which the 0 point is placed at the 26 cm mark.

**Table S4. Categories of balance with the use of the One Leg Stand Test according to time (s).**

| Time | Classification | Likert scale |
| --- | --- | --- |
| 0-14 | Very low fitness | 1 |
| 15-29 | Low fitness | 2 |
| 30-44 | Moderate fitness | 3 |
| 45-59 | High fitness | 4 |
| 60 | Very high fitness | 5 |

**Table S5. Categories of the waist circumference results (cm).**

| M | W | Classification | Likert scale |
| --- | --- | --- | --- |
| >102 | >88 | High risk | 1 |
| 99.0-102 | 86.0-88.9 | Substantial risk | 2 |
| 94.0-98.9 | 83.0-85.9 | Moderate risk | 3 |
| 90.0-93.9 | 80.0-82.9 | Low risk | 4 |
| <90 | <80 | Without risk | 5 |

Abbreviations: M, Man; W, Woman.

**Table S6. Categories of percentage of body fat according to age and sex.**

| Gender | Age range (years) | | | | | Classification | Likert Scale |
| --- | --- | --- | --- | --- | --- | --- | --- |
|  | 20-29 | 30-39 | 40-49 | 50-59 | 60-69 |  |  |
| M | ≥23.4 | ≥25.2 | ≥26.7 | ≥28.2 | ≥28.9 | Very deficient | 1 |
| W | ≥28.7 | ≥29.7 | ≥32.0 | ≥33.9 | ≥34.5 |  |  |
| M | 23.3-18.7 | 25.1-21.7 | 26.6-23.6 | 28.1-25.0 | 28.8-25.7 | Deficient | 2 |
| W | 28.6-23.6 | 29.6-24.9 | 31.9-27.5 | 33.8-30.1 | 34.4-30.9 |  |  |
| M | 18.6-14.9 | 21.6-18.5 | 23.5-20.9 | 24.9-22.4 | 25.6-23.1 | Regular | 3 |
| W | 23.5-20.1 | 24.8-21.1 | 27.4-23.7 | 30.0-26.7 | 30.8-27.6 |  |  |
| M | 14.8-10.6 | 18.4-15.0 | 20.8-17,6 | 22.3-19.5 | 23.0-20.3 | Good | 4 |
| W | 20.0-16.2 | 21.0-16.6 | 23.6-18.3 | 26.6-20.9 | 27.5-22.1 |  |  |
| M | <10.6 | <15 | <17.6 | <19.5 | <20.3 | Excellent | 5 |
| W | <16.2 | <16.6 | <18.3 | <20.9 | <22.1 |  |  |

Abbreviations: M, Man; W, Woman. ^*^Percentage body fat less than 3% and 10-13% are not recommended for men or women, respectively (31).

**Table S7. Categorization of body mass index.**

| BMI | Degree of under- or overweightness | Likert scale |
| --- | --- | --- |
| ≥40  <18.5 | Class III Obesity  Underweightness | 1 |
| 35.0-39.9 | Class II Overweightness | 2 |
| 30-34.9 | Class I Overweightness | 3 |
| 25.0-29.9 | Overweightness | 4 |
| 18.5-24.9 | Normoweight | 5 |
